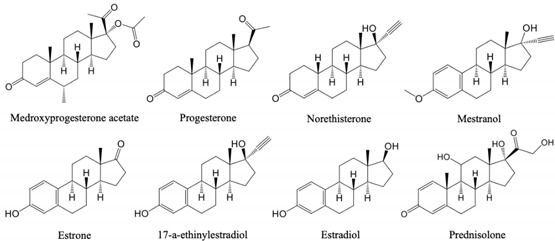# A Molecular Shape Recognitive HPLC Stationary Phase Based on a Highly Ordered Amphiphilic Glutamide Molecular Gel

**DOI:** 10.3390/nano11061574

**Published:** 2021-06-15

**Authors:** Naoki Kawamoto, Yongxing Hu, Yutaka Kuwahara, Hirotaka Ihara, Makoto Takafuji

**Affiliations:** 1 Department of Applied Chemistry and Biochemistry, Kumamoto University, 2-39-1 Kurokami, Chuo-ku, Kumamoto 860-8555, Japan; nakpmw@gmail.com (N.K.); 192d9403@st.kumamoto-u.ac.jp (Y.H.); kuwahara@kumamoto-u.ac.jp (Y.K.); 2 National Institute of Technology, Okinawa College, 905 Henoko, Nago, Okinawa 905-2192, Japan

**Keywords:** high performance liquid chromatography (HPLC), molecular gels, highly-oriented structure, stereo isomers, phenolic compounds, steroids

## Abstract

Chiral glutamide-derived lipids form self-assembled fibrous molecular gels that can be used as HPLC organic phases. In this study, HPLC separation efficiency was improved through the addition of branched amphiphilic glutamide lipids to the side chains of a terminally immobilized flexible polymer backbone. Poly(4-vinylpyridine) with a trimethoxysilyl group at one end was grafted onto the surface of porous silica particles (Sil−VP_15_, polymerization degree = 15), and the pyridyl side chains were quaternized with a glutamide lipid having a bromide group (Br***G***). Elemental analysis indicated that the total amount of the organic phase of the prepared stationary phase (Sil−VP***G***_15_) was 38.0 wt%, and the quaternization degree of the pyridyl groups was determined to be 32.5%. Differential scanning calorimetric analysis of a methanol suspension of Sil−VP***G***_15_ indicated that the ***G*** moieties formed a highly ordered structure below the phase transition temperature even on the silica surface, and the ordered ***G*** moieties exhibited a gel-to-liquid crystalline phase transition. Compared with a commercially available octadecylated silica column, the Sil−VP***G***_15_ stationary phase showed high selectivity toward polycyclic aromatic hydrocarbons, and particularly excellent separations were obtained for geometrical and positional isomers. Sil−VP***G***_15_ also showed highly selective separation for phenol derivatives, and bio-related molecules containing phenolic groups such as steroids were successfully separated. These separation abilities are probably due to multiple interactions between the elutes and the highly ordered functional groups, such as the pyridinium and amide groups, on the highly ordered molecular gel having self-assembling ***G*** moieties.

## 1. Introduction

High performance liquid chromatography (HPLC) has been widely used for the analysis and separation of organic compounds containing polymers and bio-molecules, because it is convenient and quick analytical technique. The development of HPLC stationary phases is of importance to expand its applicability in various fields. So far, various surface-modified porous silica particles having organic phases such as ionic liquid analogues [1,2,3,4,5], synthetic polymers [6,7], oligo-peptides [8,9,10], and carbon materials [11,12,13] have been developed for practical use as HPLC stationary phases. These organic phases show high selectivity toward for samples that are difficult to separate with conventional octadecylated-porous silica (ODS) in reversed-phase HPLC because of their unique interaction sites. Steric arrangement of the interaction sites is effective to enhance the selectivity of the guest molecules. The organic phase of ODS recognizes molecular hydrophobicity in reversed phase HPLC (RP-HPLC) mode which brings separation of guest molecules by partition mechanism. It has been reported that the selectivity toward polyaromatic hydrocarbons (PAHs) and their isomers can be enhanced by increasing of grafting-density of the octadecyl groups on the silica surface [14,15].

Molecular gels have been attracting significant attention because their highly oriented structures result in unique properties [16,17,18,19,20,21]. L-Glutamide derived molecules form self-assembled molecular gels in aqueous and organic media that exhibit specific functions such as supramolecular chirality and supramolecular fluorescence [22,23,24,25,26,27,28]. The amphiphilic glutamide derivatives formed nanosized fibrous aggregates based on lipid bilayer membranes with unique morphological features such as nanotubes and nanohelices [17,26,27]. In organic solvent, lipophilic glutamide derivatives formed low molecular weight organogels composed of 3-dimensional fibrous aggregate network [17,28]. These aggregates formed from the glutamide-derivatives with chromophore group showed unique optical properties such as induced circular dichroism and circularly polarized luminescence, and emission with large Stokes shift based on the highly-oriented structure with chiral arrangement [19,22,23,24,25,28].

The highly oriented structure of the self-assembled molecular gels can recognize the molecular shape of guest molecules through multiple interactions using regularly arranged functional groups. Directly immobilized lipophilic glutamide derivatives on the surface of silica microspheres recognize polycyclic aromatic hydrocarbons (PAHs), particularly the molecular planarity and the molecular length of PAHs [29,30,31,32]. We reasoned that the high selectivity of the glutamide derivatives toward guest molecules occurred through multiple interactions with the highly ordered carbonyl groups on the self-assembly. In this study, we improved the molecular orientation of the glutamide moiety by introducing it to the side chain of a flexible polymer that was immobilized on a silica surface. We then examined the molecular recognition behavior of this material as a high-performance liquid chromatography (HPLC) stationary phase.

## 2. Materials and Methods

### 2.1. Stabilization of Glutamide-Derived Lipid-Branched Polymers on Porous Silica (Sil−VP**G**_15_)

#### 2.1.1. Materials and Instruments

Porous silica microspheres (YMC*GEL, diameter = 5 µm, average pore size = 12 nm, specific surface area = 330 m^2^ g^−1^) for the stationary phase were purchased from YMC Co. Ltd. (Kyoto, Japan). All other chemicals used for the synthesis of Sil−VP***G***_15_ were purchased from Tokyo Chemical Industry Co., Ltd. (Tokyo, Japan), Fujifilm Wako Pure Chemical Co. (Osaka, Japan), Nacalai Tesque, Inc. (Kyoto, Japan), and Sigma-Aldrich Japan (Tokyo, Japan), and were purified before use as needed.

^1^H-NMR spectra were recorded at 400 MHz in CDCl_3_ at 25 °C using a JEOL JNM-LA400 spectrometer (JEOL Ltd., Tokyo, Japan). Chemical shifts (δ) are expressed in parts per million (ppm) using tetramethylsilane (TMS) as an internal standard (δ = 0.0 ppm). FT-IR spectra were obtained with a JASCO FT/IR-4100 spectrometer (JASCO Co., Tokyo, Japan). A DR PRO410-M accessory (JASCO Co.) was used for diffuse reflectance FT-IR (DRIFT) measurements. Elemental analyses were carried out on a CHN Corder MT-6 Apparatus (Yanaco Analytical Systems Inc., Kyoto, Japan). Differential scanning calorimetry was performed using DSC 7000 (Hitachi High-Tech Science Co., Tokyo, Japan). The aluminum open pan and the silver closed cell were used for solid state samples and suspension state samples respectively. The measurements were carried out the heating rate of 2 °C min^−1^ under the N_2_ gas atmosphere. The surface charge of the particles in suspension state were detected by Zetasizer Nano ZS (Malvern Panalytical Ltd., Kobe, Japan).

#### 2.1.2. Synthesis of Glutamide-Derived Lipid

**Scheme 1:**
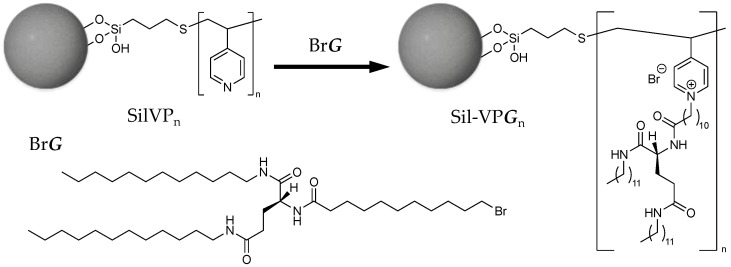
Chemical structures of Sil−VP_15_, Br***G***, and Sil−VP***G***_15_.

The glutamide-derived lipid, *N*^1^,*N*^2^-didodecyl-L-glutamide (***G***) was synthesized according to our previous report [33]. The glutamide ***G*** (4.50 g, 0.93 mmol) and 11-bromoundecanoic acid (3.42 g, 1.17 mmol) were dissolved in chloroform (300 mL), and the mixture was stirred in an ice bath. Triethylamine (3.58 mL, 25.8 mmol) and diethyl cyanophosphate (2.08 mL, 12.4 mmol) were added to the mixture, which was then stirred for 1 h in an ice bath and then for 24 h at 25 °C. The mixture was washed with 0.3 mol L^−1^ aqueous HCl solution (3 times), 5 wt% aqueous NaHCO_3_ solution (3 times), and deionized water (3 times). The solution was dried over anhydrous sodium sulfate. After removal of the sodium sulfate by filtration, the chloroform was removed by evaporation under reduced pressure. The residue was recrystallized from ethanol, and the precipitate was collected by filtration and dried in vacuo to obtain white crystals (Br***G***, Scheme 1). Yield: 6.77 g (89.7%), mp: 123.5–124.4 °C. FT-IR (KBr): 3291 cm^−1^ (ν_N–H_), 2923 cm^−1^ (ν_C–H_), 2856 cm^−1^ (ν_C–H_), 1637 cm^−1^ (ν_N–H_ (amide)), 1557 cm^−1^ (ν_N–H_). ^1^H–NMR: δ 0.86–0.90 (t, 6H, C*H*_3_), δ 1.24 (br, 48*H*, (C*H*_2_)_9_CH_3_ and BrCH_2_CH_2_(C*H*_2_)_6_), δ 1.39–1.44 (m, 2H, C(=O)CH_2_C*H*_2_), δ 1.49–1.53 (m, 4H, NHCH_2_C*H*_2_), δ 1.81–1.88 (m, 2H, C*HC*H*_2_), δ 1.92–2.10 (m, 2H, C*HCH_2_C*H*_2_), δ 2.19–2.31 (m, 2H, COC*H*_2_), δ 2.41–2.48 (m, 2H, BrCH_2_C*H*_2_), δ 3.20–3.30 (dt, 4H, NHC*H*_2_), δ 3.38–3.42 (t, 2H, BrC*H*_2_), δ 4.30–4.36 (dt, 1H, C*H), δ 6.02–6.05 (t, 1H, N*H*), δ 6.82–6.85 (t, 1H, N*H*), δ 6.99–7.01 (d, 1H, N*H*). Elemental analysis Calcd. (%) for C_40_H_78_BrN_3_O_3_: C 65.91, H 10.79, N 5.76; Found: C 64.91, H 11.76, N 5.62.

#### 2.1.3. Preparation of Poly(4-Vinylpyridine)-Grafted Porous Silica

A mixture of 4-vinylpyridine (10.51 g, 100 mmol) and 3-mercaptopropyltrimeth-oxysilane (0.98 g, 5 mmol) was stirred with nitrogen gas bubbling through the solution for 20 min. Then, 2,2’-azobis(isobutyronitrile) (105 mg, 1 wt% of 4-vinylpyridine) was added, and the mixture was stirred at 60 °C under a nitrogen atmosphere for 3 h. The obtained pale yellow viscous solution was dissolved in a small amount of chloroform, and the solution was added dropwise to diethyl ether. The precipitate that formed was washed with diethyl ether, dried in vacuo, and the product was obtained as a white powder. Yield: 7.10 g (67.6%). ^1^H-NMR: δ 0.5–0.7 (m, 2H, Si-CH_2_), δ 3.4–3.6 (s, 9H, Si-OCH_3_), δ 6.1–7.0 (m, 4H, 2- and 6-positions of pyridyl group), δ 8.1–8.7 (m, 4H, 3- and 5-positions of pyridyl group). The average polymerization degree of the poly(4-vinylpyridine) with a trimethoxysilyl reactive group at one terminus was determined to be 15 (VP_15_) from the integral ratio of the peaks of the methylene group at the γ-position of the terminal 3-mercaptopropyltrimethoxysilane and the pyridine ring.

VP_15_ (2.0 g) was dissolved in chloroform (35 mL) in a three-neck flask. Porous silica microspheres (4.0 g) were added to the solution and gently stirred at reflux for 3 days. The resulting surface-modified silica microspheres were filtered and washed with chloroform, then dried in vacuo (Sil-VP_n_, Scheme 1).

#### 2.1.4. Quaternization of Pyridyl Side Chains with Glutamide-Derived Lipid

Sil−VP_15_ (3.0 g) and Br***G*** (7.01 g) were added to toluene (30 mL), and the mixture was heated at reflux for 3 days with stirring. The product was collected by filtration through a glass filter, washed with toluene, chloroform, and methanol, in that order, and dried under reduced pressure to obtain Sil−VP***G***_15_ (Scheme 1).

### 2.2. HPLC Performance of Sil−VP**G**_15_ as the Stationary Phase

#### 2.2.1. Chemicals

All chemicals for HPLC analysis such as PAHs, phenolic derivatives, and steroids were obtained from TCI Co. Ltd. (Tokyo, Japan), Fujifilm Wako Pure Chemical Co. (Osaka, Japan), and Sigma-Aldrich Japan Inc. (Tokyo, Japan). HPLC-grade solvents were used for chromatographic separations. Octadecyl-functionalized porous silica particles (Inertsil ODS-3 packed column) were purchased from GL Science Inc. (Tokyo, Japan) and used as a reference stationary phase.

#### 2.2.2. Chromatographic Procedures

Sil−VP***G***_15_ was packed into stainless steel columns (150 mm × 4.6 mm i.d.). Liquid chromatography was performed with acrylonitrile as the mobile phase at a flow rate of 1.0 mL min^–1^ under isocratic conditions. The chromatographic system included a JASCO 2089 pump and a JASCO MD-910 UV-Vis photodiode array detector. A 5 µL sample was injected through a Rheodyne Model 7725 injector (IDEX Health & Science, LLC, Middleboro, MA USA) having a 20 µL loop. The column temperature was maintained using a column jacket. A personal computer connected to the detector with JASCO-Borwin (Ver 1.5) software was used for system control and data analysis. The separation factor (α) was determined by the ratio of retention factors (k). The retention time of D_2_O was used as the void volume (t_0_) marker. (The absorption for D_2_O was measured at 400 nm, which is actually considered to be injection shock.)

## 3. Results and Discussion

### 3.1. Characterization of Amphiphilic Glutamide-Based Molecular Gel on the Porous Silica Particle

#### 3.1.1. Grafting of Amphiphilic Glutamide-Based Molecular Gel

Poly(4-vinylpyridine) with a reactive group at one terminus (VP_n_) was prepared by telomerization of 4-vinylpyridine using 3-mercaptopropyltrimethoxysilane as a telogen.

The polymerization degree (n) was determined to be 15. In our previous report, we confirmed that the separation behavior of the polymer-immobilized porous silica was almost the same in the polymer having a degree of polymerization of 10 or more [34]. The obtained VP_15_ was immobilized on the spherical porous silica with an average particle size of 5 µm and an average pore size of 12 nm, which is commonly used for HPLC packing material. The grafting amount of VP_15_ on the surface of the porous silica was determined by elemental analysis to be 17.2 wt%.

**Figure 1:**
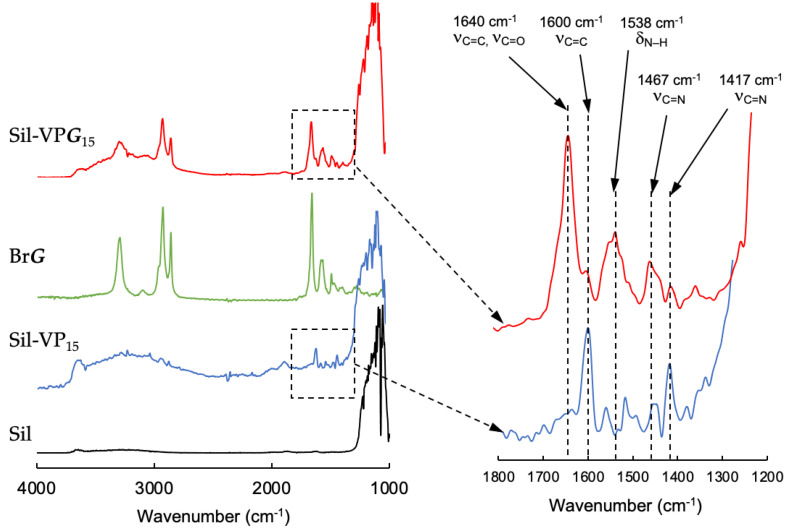
Diffuse reflectance FT−IR spectra of Sil, Sil−VP_15_, Br***G***, and Sil−VP***G***_15_.

The quaternization reaction of the pyridyl side chain was confirmed by the FT-IR spectrum (Figure 1), in which the peaks of the C=C and C=N stretching vibrations of the pyridine ring shifted from 1600 to 1640 cm^−1^ and from 1417 to 1467 cm^−1^, respectively [35,36]. In addition, a C–H stretching band at approximately 2850–2950 cm^−1^ that was due to the long alkyl chain of the ***G*** moiety appeared after quaternization.

Elemental analysis of the polymer-grafted porous silica (Table 1) showed an increase in the amount of the organic phase accompanied by an increase in the C/N ratio from 6.1 to 8.7. From these results, the percentage quaternization of the pyridyl groups with Br***G*** was calculated as 32.5%. Because the pyridinium salt after quaternization is cationic, success of the quaternization reaction was also determined by measuring the zeta potential at the particle interface, which confirmed that the particle interface of Sil−VP***G***_15_. was more positively charged than Sil−VP_15_.

**Figure 2:**
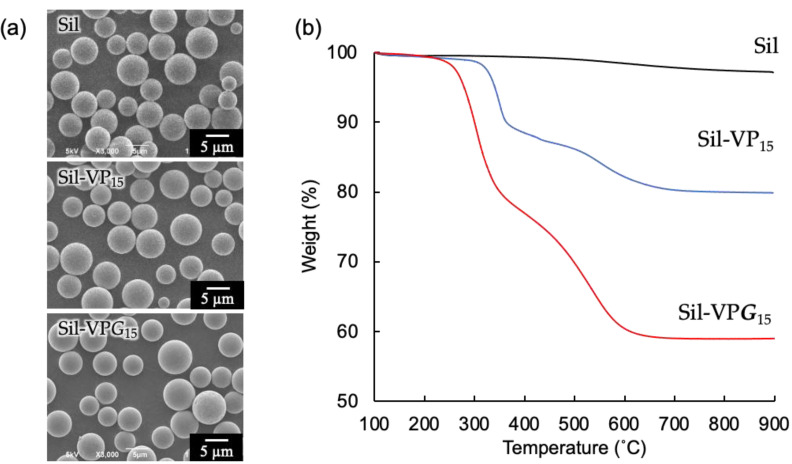
(**a**) SEM images and (**b**) DSC thermograms of Sil, Sil−VP_15_, and Sil−VP***G***_15_. The particles were coated with osmium for the SEM observations. Heating rate for DSC: 10 °C min^−1^.

Thermogravimetric analysis of Sil−VP_15_ and Sil−VP***G***_15_ (Figure 2), in which the amount of the organic phase increased from 17.2 wt% before quaternization to 38.0 wt% after quaternization, supported this conclusion. SEM images of the porous silica particles before (Sil) and after polymer grafting (Sil−VP_15_) and quaternization (Sil−VP***G***_15_) showed no noticeable change in appearance of the spherical shape, which indicated that the polymer grafting and quaternization had occurred on the surface of the porous silica particles.

#### 3.1.2. Evaluation of Amphiphilic Glutamide-Based Molecular Gels on the Silica Surface

The obtained Sil−VP***G***_15_ could be dispersed in organic solvents such as methanol and acetonitrile. The surface charge of the porous silica particles was −11.4 mV in methanol, which changed to +7.7 after VP_15_ grafting (Sil−VP_15_) and +32.8 after quaternization with Br***G*** (Sil−VP***G***_15_). These results suggest that the surface of Sil−VP***G***_15_ is amphiphilic and positively charged.

**Figure 3:**
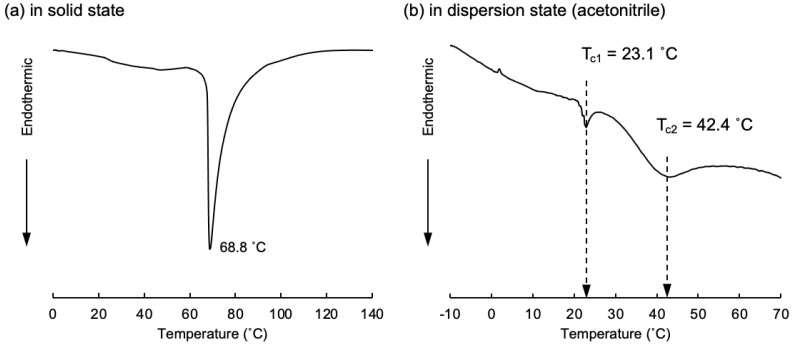
DSC thermograms of Sil−VP***G***_15_ (**a**) in the solid state and (**b**) in the suspension state (acetonitrile). Heating rate: 2 °C min^−1^ for the solid state and 1 °C min^−1^ for the suspension state.

The DSC thermogram of Sil−VP**G**_15_ in the solid state (Figure 3a) showed an endothermic peak at 68.8 °C, indicating that the **G** moiety of the side chain can form a crystalline structure on the surface of the silica. Since the melting point of Br**G** (123.5–124.4 °C) is higher than 68.8 °C, it is likely that the crystallization of the G moiety is perturbed by the polymer backbone. For Sil−VP**G**_15_ dispersed in acetonitrile, phase transition behavior was observed at 42.4 °C with a pre-transition at 23.1 °C (Figure 3b). According to our previous work, the amphiphilic glutamide derivative with a pyridinium terminal group showed phase transitions at 33 °C (pre-transition) and 45 °C (main transition), which were due to the rearrangement of intermolecular hydrogen bonds between amide bonds and the gel-to-liquid crystalline phase transition of the G moiety, respectively [17]. And the lipophilic glutamide derivative showed a gel-to-dispersion (solution) phase transition in organic solvents [17]. The aggregation morphology and the orientation structure of amphiphilic and lipophilic **G**-derived molecules are strongly affected by the solvent [17,26,27,28]. It is notable that the highly ordered structure of the **G** moiety was maintained while on the polymer side chain of the terminally grafted polymer on the surface of the silica substrate

### 3.2. HPLC Performance of Amphiphilic Glutamide-Based Molecular Gel

#### 3.2.1. Evaluation of Sil−VP***G***_15_ as an HPLC Stationary Phase

**Figure 4:**
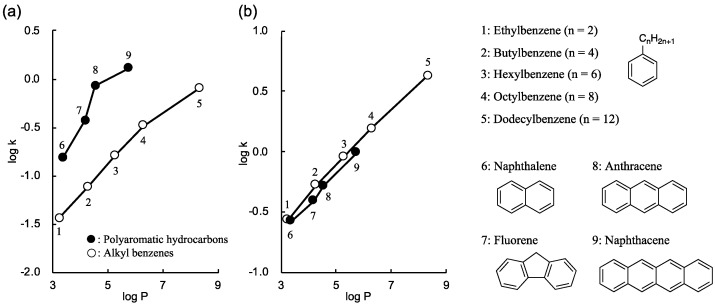
Relationships of log k and log P for (**a**) Sil−VP***G***_15_ and (**b**) ODS. Mobile phase: Acetonitrile. Flow rate: 1.0 mL min^−1^. Detection wavelength: 254 nm. Column temp.: 25 °C.

The ***G*** moiety of Sil−VP***G***_15_ possesses hydrophobic long alkyl chains and amide bonds, which were introduced to the side chain of the poly(vinylpyridine) through a quaternization reaction. The amphiphilic side chains of the ***G*** moiety formed highly ordered structures on the silica surface even in polar organic solvents such as acetonitrile. Therefore, Sil−VP***G***_15_ was evaluated as a packing material in RP-HPLC retention studies using alkylbenzenes and PAHs as analytes. Octadecylated porous silica microspheres (ODS), which are commonly used for the stationary phase in RP-HPLC, were used as a reference. Figure 4 shows the relationship between log k (k = (t − t_0_)/t_0_, where k is the retention factor, t is the retention time of the elute, and t_0_ is the void volume) and log P (P is the 1-octanol–water partition coefficient) for ODS and Sil−VP***G***_15_. The log k and log P plots of the alkylbenzenes and PAHs in ODS were almost identical. However, Sil−VP***G***_15_ showed a higher retention for PAHs than for alkylbenzenes. These results provided evidence that the ODS stationary phase can recognize only the hydrophobicity of the analytes, but Sil−VP***G***_15_ has specific interaction sites as well as molecular hydrophobicity for the retention of PAHs.

We had previously reported that carbonyl group-containing stationary phases recognize the aromaticity of the elutes through carbonyl π−benzene π interactions [37,38,39]. Particularly highly ordered carbonyl groups can recognize the molecular planarity and the molecular length of PAHs as well. Sil−VP***G***_15_ also probably recognizes the π−electrons of the PAHs through multiple interactions with the ordered carbonyl groups on the oriented ***G*** moieties.

#### 3.2.2. Temperature Dependence of the Separation Factors for PAH Isomers

**Figure 5:**
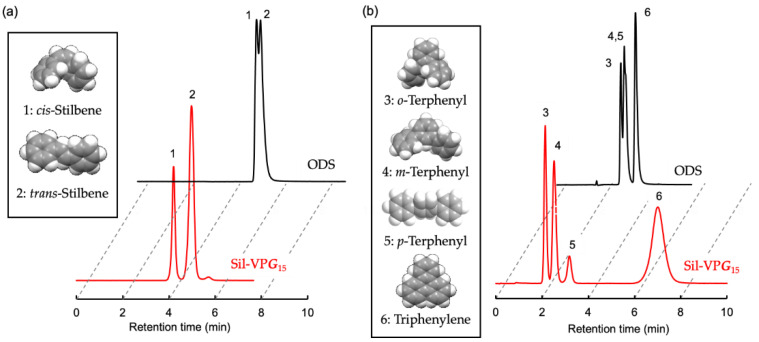
Chromatograms for (**a**) a mixture of *cis*-stilbene and *trans*-stilbene and (**b**) triphenylene and *o*-, *m*-, and *p*-terphenyls using Sil−VP***G***_15_ and ODS as stationary phases. Mobile phase: Acetonitrile. Column temp.: 25 °C. Flow rate: 1.0 mL min^−1^.

We previously reported that self-assembling molecule-immobilized stationary phases, in which the glutamide derivative is directly grafted to the porous silica particles through a siloxane linkage, show high selectivity toward PAHs [29,30,31,32]. Compared with these stationary phases, the self-assembling ***G*** moiety may have relatively less perturbation from the silica surface because the flexible polymer chain is branched. Figure 5 shows the chromatograms for *cis*− and *trans*−stilbene; *o*−, *m*−, and *p*−sterphenyl; and triphenylene using Sil−VP***G***_15_ and a commercially available ODS. Sil−VP***G***_15_ showed baseline separations for these isomers whereas the ODS did not.

The retention and separation factors of these molecules using Sil−VP***G***_15_ and ODS at 0 °C and 50 °C are summarized in Table 2. The isomers were eluted earlier using the Sil−VP***G***_15_ stationary phase than they were using the ODS stationary phase, which is probably due to the amphiphilic nature of organic phase. However, Sil−VP***G***_15_ showed extremely high selectivity toward these isomers at temperatures below (0 °C) and above (50 °C) its phase transition. These results indicate that the ***G*** moiety recognizes molecular shape even in a disordered state, and this is enhanced through orientation of the ***G*** moiety.

**Figure 6:**
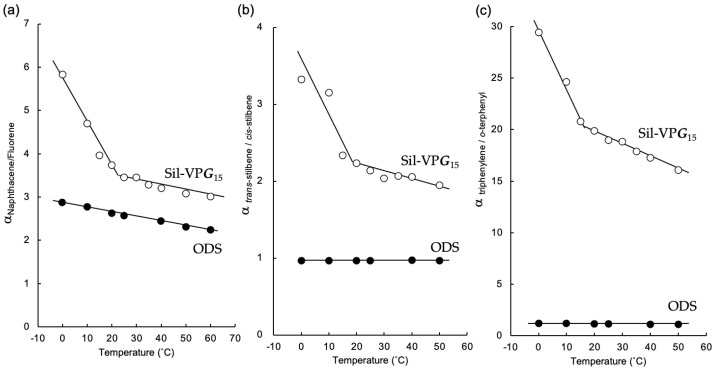
Temperature dependence of the separation factors for (**a**) naphthacene/fluorene, (**b**) *trans*-stilbene/*cis*-stilbene and (**c**) triphenylene/*o*-terphenyl using Sil−VP***G***_15_ and ODS as stationary phases. Mobile phase: Acetonitrile. Column temp.: 25 °C. Flow rate: 1.0 mL min^−1^.

The temperature dependency of the separation factors of naphthacene/fluorene, *trans*-stilbene/*cis*-stilbene, and triphenylene/*o*-terphenyl are shown in Figure 6. The α value increased with decreasing temperature, and increased sharply below around 20 °C, which corresponds to the temperature of the pre-phase transition. In each case, planar molecules eluted later than non-planar molecules. This may be an effect of the orientation of the ***G*** moiety.

#### 3.2.3. Selective Separations of Aromatic Compounds Having Hydrophilic Functional Groups

**Figure 7:**
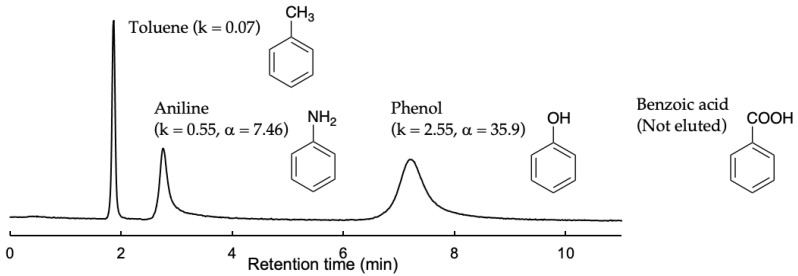
Chromatogram of toluene, aniline, phenol, and benzoic acid using Sil−VP***G***_15_. Mobile phase: Acetonitrile. Flow rate: 1.0 mL min^−1^. Temperature: 25 °C.

Monosubstituted benzenes such as phenol, aniline, and benzoic acid, were examined for chromatographic separation using Sil−VP***G***_15_. As shown in Figure 7, the elution order was toluene (methyl group) < aniline (amino group) < phenol (hydroxyl group) < benzoic acid (carboxylic acid group). Benzoic acid probably did not elute as a result of electrostatic interaction with the pyridinium group on Sil−VP***G***_15_. All monosubstituted benzenes with a polar group eluted later than toluene, indicating that retention was determined by a hydrophilic interaction (HILIC)/RP mixed-mode mechanism. It is noticeable that the organic phase of the Sil−VP***G***_15_ recognized not only hydrophilic groups but also hydrophobic groups due to its amphiphilic nature.

The positional isomers of monosubstituted phenols such as methyl phenols, dihydroxy benzenes, and aminophenols were also examined using Sil−VP***G***_15_. As shown in Table 3, the elution order of mono-substituted phenols was methylphenols < aminophenols < dihydroxybenzenes, which was the same as the order of the substitutional group of the monosubstituted benzenes described above. As expected, the Sil−VP***G***_15_ column recognized positional isomers of these substituted phenols.

#### 3.2.4. Application of Sil−VP***G***_15_ Column to Separations of Steroids

Steroids are amphiphilic compounds comprised of a skeleton of three hexagonal carbon rings and one pentagonal carbon ring, to which various functional groups such as hydroxyl are attached. Table 4 shows the retention and separation factors of eight steroids using a Sil−VP***G***_15_ column compared with those obtained via an ODS column. Sil−VP***G***_15_ was able to separate the steroids based on their number of hydroxyl groups. The steroid (chemical structures are mentioned in the footnote of Table 4) elution order was: no hydroxyl groups (medroxyprogesterone acetate and progesterone) < one hydroxyl group (norethisterone, mestranol, and estrone) < two hydroxyl groups (17-α-ethinylestradiol and estradiol) < three hydroxyl groups (prednisolone). The separation factors increased significantly as the number of hydroxyl groups increased.

## 4. Conclusions

Amphiphilic glutamide was successfully introduced as a self-assembling molecular gel to the side chain of a terminally grafted poly(4-vinylpyridine) on the surface of porous silica microspheres. The ***G*** moieties formed a highly ordered structure and showed phase transitions at 23.1 °C (pre-phase transition) and at 42.4 °C (main phase transition) in an acetonitrile suspension even on the surface of a solid substrate. Compared with a conventional ODS column, Sil−VP***G***_15_ showed high selectivity toward PAHs, and particularly excellent separations were obtained for geometrical and positional isomers such as stilbenes and triphenylenes. This separation ability is probably due to multiple interactions between the elutes and functional groups on the highly oriented ***G*** moiety of the organic phase, such as pyridinium and amide bonding. Furthermore, Sil−VP***G***_15_ recognized substituted benzenes in the order of toluene (methyl group) < aniline (amine group) < phenol (hydroxyl group) < benzoic acid (carboxylic acid). Highly selective separations were obtained for positional isomers of phenolic compounds such as methyl phenols, dihydroxy benzenes, and aminophenols. We also demonstrated the separation of eight steroid compounds having different chemical structures and different numbers of hydroxyl groups. Isolation and purification of biological molecules such as peptides, proteins, saccharides and nucleosides are indispensable technology in the fields of proteomics, genomics, and glycomics [40,41,42]. Further study for the separation of bio-related molecules using Sil−VP***G***_15_ is currently underway and will be reported in the future.

## Figures and Tables

**Table 1:** Elemental analysis of Sil−VP_15_ and Sil−VP***G***_15_.

Stationary Phase		C%	H%	N%	C/N	Organic Phase (wt%)
Sil−VP_15_	Found	13.4	1.73	2.18	6.13	17.2
	Calcd ^1^	15.7	1.37	2.56	6.13	
Sil−VP***G***_15_	Found	27.6	4.48	3.18	8.68	38.0
	Calcd ^2^	28.7	3.89	3.31	8.67	

^1^ Calcd. for C_108_H_112_SN_15_ (17.2 wt% of organic phase). ^2^ Calcd. for C_303_H_494_N_420_O_240_S_32_ (38.0 wt% of organic phase).

**Table 2:** Retention factors (k) and separation factors (α) of stilbene and terphenyl isomers using Sil−VP***G***_15_ and ODS.

Elutes	Sil−VP*G*_15_				ODS			
	0 °C		50 °C		0 °C		50 °C	
	k	α	k	α	k	α	k	α
*cis*-Stilbene	0.21	–	0.05	–	0.61	–	0.26	–
*trans*-Stilbene	0.68	3.24	0.09	1.80	0.55	0.90 (1.11)	0.22	0.85 (1.18)
*o*-Terphenyl	0.28	–	0.09	–	0.70	–	0.31	–
*m*-Terphenyl	0.76	2.71	0.19	2.11	0.84	1.20	0.35	1.13
*p*-Terphenyl	1.74	6.21	0.34	3.78	0.94	1.34	0.37	1.19
Triphenylene	8.36	29.86	1.39	15.44	1.36	1.94	0.51	1.65

Mobile phase: Acetonitrile. Flow rate: 1.0 mL min^−1^.

**Table 3:** Retention factors (k) and separation factors (α) of disubstituted benzenes using Sil−VP***G***_15_ and ODS.

Elutes	Sil−VP*G*_15_		
	k	α	α (vs. Phenol)
Phenol	2.55		–
2-Methyl phenol (*o*-isomer)	2.37	–	0.93
3-Methyl phenol (*m*-isomer)	2.18	0.92	0.86
4-Methyl phenol (*p*-isomer)	2.66	1.22	1.04
1,2-Dihydroxy benzene (*o*-isomer)	24.2	–	9.49
1,3-Dihydroxy benzene (*m*-isomer)	27.4	1.13	10.75
1,4-Dihydroxy benzene (*p*-isomer)	34.4	1.42	13.49
2-Aminophenol (*o*-isomer)	12.4	–	4.86
3-Aminophenol (*m*-isomer)	16.8	1.35	6.59
4-Aminophenol (*p*-isomer)	24.2	1.95	9.49

Mobile phase: Acetonitrile, Flow rate: 1.0 mL min^−1^. Temperature: 25 °C.

**Table 4:** Retention factors (k) and separation factors (α) of steroids using Sil−VP***G***_15_ and ODS.

Elutes ^1^	Sil−VP*G*_15_		ODS	
	k	α	k	α
Medroxyprogesterone acetate	0.07	0.27	0.48	0.71 (1.42)
Progesterone	0.26	1.00	0.68	1.00
Norethisterone	0.81	3.11	0.26	0.38 (2.62)
Mestranol	1.16	4.48	0.39	0.57 (1.74)
Estrone	4.06	15.7	0.22	0.32 (3.09)
17-α-Ethinylestradiol	12.3	47.6	0.14	0.21 (4.86)
Estradiol	14.2	54.7	0.29	0.43 (2.34)
Prednisolone	22.8	87.7	0.12	0.18 (5.67)

Mobile phase: Acetonitrile. Flow rate: 1.0 mL min^−1^. Temperature: 25 °C. ^1^ Chemical structures of elutes are as follows.